# Implementation of two policies to extend maternity leave and further restrict marketing of breast milk substitutes in Vietnam: a qualitative study

**DOI:** 10.1093/heapol/czab116

**Published:** 2021-09-18

**Authors:** Denise Diaz Payán, Neha Zahid, Jeffrey Glenn, Ha TT Tran, Tran Thi Thu Huong, Corrina Moucheraud

**Affiliations:** Department of Public Health, School of Social Sciences, Humanities and Arts, University of California Merced, 5200 N Lake Road, Merced, CA 95343, USA; Department of Public Health, School of Social Sciences, Humanities and Arts, University of California Merced, 5200 N Lake Road, Merced, CA 95343, USA; Department of Public Health, College of Life Sciences, Brigham Young University, 4103 LSB, Provo, UT 84602, USA; Research and Training Centre for Community Development (RTCCD), No 39 Lane 255, Vong Street, Hai Ba Trung District, Hanoi, Vietnam; Research and Training Centre for Community Development (RTCCD), No 39 Lane 255, Vong Street, Hai Ba Trung District, Hanoi, Vietnam; Department of Health Policy and Management, University of California Los Angeles, Fielding School of Public Health, 650 Charles E. Young Dr. South, Los Angeles, CA 90095-1772, USA

**Keywords:** policy implementation, breastfeeding, maternal and child health, nutrition, qualitative research, policy process

## Abstract

Policy research can reveal gaps and opportunities to enhance policy impact and implementation. In this study, we use a theoretically informed qualitative approach to investigate the implementation of two policies to promote breastfeeding in Vietnam. We conducted semi-structured interviews with national and local policy stakeholders (*n* = 26) in 2017. Interviews were audio-recorded, transcribed verbatim and then translated to English by certified translators. Transcript data were analysed using an integrated conceptual framework of policy implementation. Respondents identified several positive outcomes resulting from implementation of an extended maternity leave policy (Labour Code No. 10/2012/QH13) and further restrictions on marketing of breast milk substitutes (Decree No. 100/2014/ND-CP). Decree No. 100, in particular, was said to have reduced advertising of breast milk substitutes in mass media outlets and healthcare settings. Key implementation actors were national-level bureaucratic actors, local organizations and international partners. Findings reveal the importance of policy precedence and a broader set of policies to promote the rights of women and children to support implementation. Other facilitators were involvement from national-level implementing agencies and healthcare personnel and strength of government relationships and coordination with non-governmental and international organizations. Implementation challenges included insufficient funding, limited training to report violations, a cumbersome reporting process and pervasive misinformation about breast milk and breast milk substitutes. Limited reach for women employed in the informal labour sector and in rural communities was said to be a compatibility issue for the extended maternity leave policy in addition to the lack of impact on non-parental guardians and caretakers. Recommendations to improve policy implementation include designating a role for international organizations in supporting implementation, expanding maternity protections for all working women, building local-level policy knowledge to support enforcement, simplifying Decree No. 100 violation reporting processes and continuing to invest in interventions to facilitate a supportive policy environment in Vietnam.

**Key messages**

- This study uses an integrated theoretical framework to examine the implementation of an extended maternity leave policy and further restrictions on the marketing of breast milk substitutes (Decree No. 100) in Vietnam.
- Key facilitators to implementing new policies to promote breastfeeding include policy precedence, involvement from national-level implementing agencies and healthcare personnel, strength of government relationships and coordination with non-governmental and international organizations.
- Important policy implementation challenges were insufficient funding, a lack of communication between national and regional authorities, limited training among frontline workers to report violations (Decree No. 100), and myths and misinformation about infant and young child feeding.
- Strategies to improve policy implementation may include designating a policy implementation role for non-governmental organizations, expanding maternity protections, simplifying reporting violation processes and continuing to invest in interventions to facilitate a supportive policy environment for maternal and child health in Vietnam.

## Introduction

Implementing evidence-based policies to promote optimal infant and young child feeding (IYCF) practices (e.g. exclusive breastfeeding, complementary feeding) is a critical strategy for improving child health worldwide. Globally, an estimated half a million to 800 000 young children die each year due to preventable conditions (e.g. diarrhoea and pneumonia) from suboptimal breastfeeding practices (Black *et al.*, 2013; Walters *et al.*, 2019). Poor IYCF practices contribute to childhood malnutrition-related conditions—including stunting or wasting due to undernutrition or obesity due to overnutrition—and cognitive decline in early childhood, all of which have detrimental long-term health impacts for individuals and are associated with economic disadvantage for regions and countries (Horta *et al.*, 2015; Rollins *et al.*, 2016; Victora *et al.*, 2016).

Policy interventions to support breastfeeding include expanding paid maternity leave to promote exclusive breastfeeding and breastfeeding duration (Chai *et al.*, 2018; Keats *et al.*, 2021) and full implementation of the International Code of Marketing of Breast-milk Substitutes (the Code, adopted by the World Health Assembly in 1981) to limit advertising and promotion of breast milk substitutes, feeding bottles and artificial teats (Rollins *et al.*, 2016; World Health Organization, 2017). Stronger efforts are needed to strengthen policy implementation and effectiveness of policies to increase breastfeeding rates (Ching *et al.*, 2021; World Health Organization, 2020).

Policy research can provide valuable information to scale up effective interventions and promote the implementation of evidence-based policies to improve maternal and child health outcomes (Menon and Thow, 2017). Implementation research, in particular, can help researchers and practitioners understand local context and policy considerations, anticipate whether policies and programmes have an intended impact and identify key facilitators and barriers to implementation (Ghaffar *et al.*, 2020; Nguyen *et al.*, 2020; Rasanathan, 2015; Walt *et al.*, 2008). A lack of information on policy compliance is a noted limitation for maternal and child health policy effectiveness research (Chai *et al.*, 2018). Further, existing health policy implementation research in low- and middle-income countries (LMICs) is scarce and lacking theoretical conceptualization (Erasmus *et al.*, 2014), despite being a critical phase of the policymaking process (Allen *et al.*, 2020) that comes after policy development and adoption.

Ongoing efforts to assess the implementation of IYCF policies include the World Breastfeeding Trends Initiative, which tracks information on policies and programmes through interviews and government documents (Gupta *et al.*, 2013). Existing IYCF policy research has mostly examined policy environments, policy change and stakeholder influence in Southeast Asia, South Asia and East Africa (Menon and Thow, 2017), with additional work needed on policy implementation and enforcement (Harris *et al.*, 2017).

Between 2009 and 2014, the IYCF programme by Alive and Thrive and its partners was implemented in three countries (Bangladesh, Ethiopia and Vietnam), with funding from the Bill and Melinda Gates Foundation (Menon *et al.*, 2013). Programme activities varied by country with four primary components: interpersonal counselling, mass media campaigns, community mobilization and policy advocacy. Programme evaluation findings suggest that the programme led to improvements in IYCF behaviours and outcomes in Vietnam and Bangladesh (Kim *et al.*, 2018; Menon *et al.*, 2016; Nguyen *et al.*, 2014). A mixed-method sustainability study of the Alive and Thrive Initiative in these countries 2 years after funding ceased found a sustained impact for IYCF interpersonal counselling activities and health worker knowledge but a perceived decline in other programme components (Moucheraud *et al.*, 2020). In Vietnam, Alive and Thrive policy advocacy activities resulted in the following policy changes: an extended maternity leave policy in the Labour Code and further restrictions on the marketing of breast milk substitutes (Harris *et al.*, 2017).

This article uses a novel integrated theoretical framework of the policy implementation process (Bullock *et al.*, 2021) to (1) identify policy actors involved in implementing policy changes to extend maternity leave and further restrict marketing of breast milk substitutes in Vietnam and (2) examine determinants of implementing these two policies. Examining the implementation of these maternal and child health policies can help us to better understand the long-term impact and sustainability of policy advocacy interventions as well as inform strategies to enhance implementation.

## Methods

In this study, we used a theoretically informed qualitative approach to conduct interviews with national and local policy actors to examine the implementation of two policies to advance maternal and child health in Vietnam.

### Study setting and policy context

Vietnam is a lower middle-income country with a population of 96.5 million residents and a gross domestic product of $270 billion in 2019 (The World Bank, 2021). The country’s exclusive breastfeeding rate was 24% in 2014, falling short of the minimum 50% exclusive breastfeeding target rate set by the World Health Assembly (WHO, UNICEF, 2014). An estimated 3572 child deaths per year are attributed to suboptimal breastfeeding rates (Alive and Thrive, 2021). Child overnutrition—due in part to the country’s economic transition and food environment shifts—has also increasingly become a vital health issue, resulting in an increased risk for childhood dental caries and obesity (Baker *et al.*, 2016; Huang *et al.*, 2019).

Since 2010, Vietnam’s policy successes to promote breastfeeding include ratification of further restrictions on marketing of breast milk substitutes (Decree No. 100) and an extended maternity leave policy in the Labour Code. The first policy, Decree No. 100, was an amendment to Decree 21 (adopted 2006), which banned advertisement of breast milk substitutes for children <12 months of age. In 2012, the National Assembly voted in favour of Decree No. 100, which included the prohibition of advertising breast milk substitutes for children <24 months in addition to other marketing regulations to promote recommended IYCF practices. In 2014, additional restrictions were added to prohibit marketing and use of feeding bottles, teats and pacifiers (Decree No. 100/2014/ND-CP). The extended maternity leave policy (Labour Code No. 10/2012/QH13) was passed in 2013 by the National Assembly and is an amendment to the Labour Code, extending paid maternity leave from 4 to 6 months for mothers working in the formal sector (Harris *et al.*, 2017; Nguyen *et al.*, 2020).

### Data collection

We conducted semi-structured key informant interviews with a purposive sample of national and local IYCF stakeholders in 2017. National and local stakeholders were first identified based on their involvement in IYCF policy development and implementation. This list was revised in discussion with Alive and Thrive programme and government partners. Then, local participants were asked to identify others with the knowledge of Alive and Thrive and IYCF programme activities or who were involved in the programme’s implementation.

The interview tool included questions on stakeholder perspectives of Alive and Thrive Initiative programme success, implementation facilitators and challenges, adaptations, replication, leadership capacity, existing partnerships, service quality, funding sources, monitoring and evaluation processes, and best practices (Supplementary data). Pertinent to policy implementation, the tool included broad questions about successes, challenges and facilitators associated with the Alive and Thrive Initiative with probes on adoption and implementation of new IYCF policies.

The tool was initially developed in English, with input from the research team in Vietnam who translated the final version and verified the output using back-translation. Interviews were audio-recoded and transcribed verbatim in Vietnamese. Additional data collection details are available elsewhere (Moucheraud *et al.*, 2020).

For this study, all national key informant interview transcripts (*n* = 13) and a sub-set of local stakeholder transcripts (*n* = 13) were translated into English by certified translators. In the parent study, a total of 105 interviews were conducted with local stakeholders from six provinces where the Alive and Thrive funded intervention (‘Little Sun’) was implemented. Nearly all were audio-recorded with consent, and only five local stakeholders declined to be recorded (a note-taking protocol was employed instead). We randomly selected at least two local stakeholder interview transcripts from five provinces (Bắc Ninh, Cà Mau, Quảng Ngãi, Thái Bình and Thanh Hóa) for translation and inclusion in this study. Interviews from the sixth province (Da Nang) were not included because they did not include policy implementation data.

National key informants mostly consisted of maternal and child health policymakers and experts from the public sector. Two were from a donor agency or development partner and three from a private sector, not-for-profit organization. Local stakeholders were public sector employees, including district government officials, government representatives and health workers.

### Data analysis

Transcripts were uploaded to Dedoose, a qualitative data management software program used to organize interview data for coding and analysis (2020). We used a combination of inductive and deductive methods to identify principal themes and codes (sub-themes) (Miles and Huberman, 1994).

First, we relied on an integrated theoretical framework of the policy implementation process to guide our analysis. The use of this integrated framework addresses prior critiques of policy implementation research in LMICs that note a lack of theorization as the basis for this work (Erasmus *et al.*, 2014). The selected framework draws from the fields of public policy, implementation science and knowledge translation. The framework includes the following components: external environment/policy context, determinants of implementation, policy instruments and strategies, policy actors, implementation processes, and outputs and outcomes (Bullock *et al.*, 2021). Based on the framework, the principal theme of policy actors was divided into five codes: political actors, bureaucratic actors, special interests, experts and other. Policy implementation determinants are defined as the independent variables that affect implementation outcomes with eight determinants in the framework (Bullock *et al.*, 2021). We identified other possible determinants of IYCF policy implementation based on a literature review of this topic (Avula *et al.*, 2017; Godakandage *et al.*, 2017; Harris *et al.*, 2017; Puri *et al.*, 2017; Rasheed *et al.*, 2017; Thow *et al.*, 2017) and included these as codes.

Next, research staff tested the pilot codebook with five transcripts and identified emergent codes within principal themes. The final codebook included the following 13 principal themes: policy instruments, policy actors, characteristics of evidence-informed policy or practice, vertical administration and thickness of hierarchy, networks/inter-organizational relationships, implementing agency responses, attributes and responses of those affected by the policy, external environment, facilitators, challenges or barriers, outcomes, policy advocacy and recommendations. Each theme contained various codes.

A research staff member coded all transcripts using the codebook and identified questions to discuss with the lead author. The lead author reviewed all coded content. Select summaries were reviewed by the analysis team to extract key findings.

## Results

Respondents provided detailed information about implementation of Decree No. 100 and the extended maternity leave policy in the Labour Code. Many respondents lauded Decree No. 100 and the extended maternity leave policy for prioritizing the rights of women and children. The policies’ focus on improving the health and well-being of expectant mothers, new mothers and infants was described in positive terms, as illustrated by the following quote by a respondent from the Ministry of Health: ‘The policies supported by Alive and Thrive primarily target the health of mothers and children, but they are also largely linked with young children’. A local policymaker from Thanh Hóa commented on the marked improvement of their exclusive breastfeeding rate:

In the past, Thanh Hóa was one of the provinces with the lowest rates of exclusive breastfeeding in the first 6 months. Only about 5%. Now, according to the Nutrition Institute’s evaluation before the program ended, at 20%. Recently, it increased to about 30%.

Several national-level actors spoke of the importance of enacting and implementing policy to support optimal IYCF programmes and practices, including the Alive and Thrive Initiative programme components that had ended or waned once external funding ceased. A respondent spoke about the importance of policy adoption and implementation to sustain these efforts:

Regarding sustainability, the project activities shall be linked with state management agencies and integrated with practical and feasible policies. Examples are the Decree 100 and decree on a longer parental leave. These provide clear benefits to mothers and women. (Ministry of Health)

One participant described these policies as synergistically creating an enabling environment for breastfeeding:

The Labour Code provides a framework for women to stay at home to breastfeed for six months and receive full pay without any fear of job loss. But in order to help the mother to exclusively breastfeed her baby without the interference of external factors, such as the advertising of dairy companies or misleading healthcare practitioners…there must be a change in the awareness of both the health sector and the whole society, synergized by the Law on Advertising. (National Assembly)

Decree No. 100 was generally discussed using positive terms, as illustrated by the following quote from a policy stakeholder in the Ministry of Health who described the law as a milestone toward improving nutrition: ‘This is an important policy related to the standards, advertising, communication, awareness, and responsibilities of mothers and their families, responsibilities of healthcare facilities, health workers, and businesses to inform about mothers’ use of nutritional products’.

Implementation of Decree No. 100 was said to contribute to changes in the advertising environment and reduce the quantity of advertisements in various settings (i.e. mass media outlets and healthcare settings). It was also said to limit the visibility of the breast milk substitute industry. In the words of a policy actor in the National Assembly, ‘The way dairy companies advertise on TV and in newspapers has changed completely. Advertisements in the mass media has decreased and panels and posters in hospitals have almost disappeared completely’.

The extended maternity leave policy was similarly described in positive terms and believed to prioritize the nutrition of new mothers and young children, as illustrated by a quote from a respondent in the Ministry of Health, ‘This is an important policy, benefiting the health of mothers and their newborn babies while giving mothers more time to take care of and breastfeed their children’. Respondents said extending leave from 4 to 6 months helped to promote exclusive breastfeeding and gave new mothers sufficient time to recover without concern about job loss, as demonstrated by the following excerpt by a participant from the National Assembly: ‘In order to improve the future generations in Vietnam, it was necessary to increase the breastfeeding rate, which required a paid maternity leave extension so that the mother can stay at home and come back without worrying about losing their job’.

### Policy actors

National-level bureaucratic actors identified as being involved in policy implementation included the Ministry of Health; Ministry of Information and Communications; Ministry of Labor, Invalids, and Social Affairs (MOLISA); National Assembly; Women’s Association (Vietnam Women’s Union) and Vietnam General Federation of Labour. Specific departments mentioned as central to implementation were the Department of Maternal and Child Health, Department of Legal Affairs and National Institute of Nutrition. Special interest groups and experts who were frontline workers in bureaucracies or local implementing agencies consisted of community-level partners, healthcare organizations, medical workers, Peoples’ Councils and local Departments of Health.

Alive and Thrive was described as an integral partner, specifically as a champion for implementation and an intermediary between policy stakeholders who provided evidence, facts, documents and tools to support policymaking and implementation. Alive and Thrive, UNICEF, WHO and Save the Children were said to provide important technical support for implementation. Specifically, Save the Children was seen as influential in enforcing the extended maternity leave policy by providing breastfeeding health education for women.

Industry organizations were not explicitly described as key policy actors. Respondents only commented about the impact of ongoing lobbying efforts by the breast milk substitute industry and interactions between the industry and implementing agencies.

### Policy implementation determinants

Next, we provide results for the determinants of policy implementation. Qualitative data mapped onto six of eight policy implementation determinants in the integrated framework: characteristics of the evidence-informed policy or practice, vertical public administration and thickness of hierarchy, networks/inter-organizational relationships, implementing agency responses, attributes and responses of those affected by the policy, and external environment or policy context. We do not report on policy formulation or timing/sequence due to a lack of data on these specific determinants. Table 1 provides a list of the policy implementation determinants, key findings and supporting quotes.

**Table 1.** Determinants of breastfeeding policy implementation (PI) in Vietnam (2017): PI determinant, key finding and supporting quotes

PI determinant^a^	Key findings	Supporting quote(s)
Characteristics of the evidence-informed policy or practice	Value of policy precedence for further restrictions on marketing of breast milk substitutes (Decree No. 100)	‘Decree No. 100 has mentioned all the requirements…it does not need any more documents. The sanctions, fines and administrative violations are mentioned in other decrees. So when the decree is issued, it can be implemented immediately without any guidance, not to mention that we previously had a precursor of Decree 21.’ (Alive & Thrive Vietnam)
	Lack of compatibility between the extended maternity leave policy and local contexts/realities (e.g. caregiving situations and distance between work and home environments)	‘The associations themselves are places where, for example, working mothers who have given birth are not allowed to take leave for the first three months, and thus childcare is taken over by babysitters or grandparents. I think we have to review our policies and regimes to know whether they are practical.’ (Ministry of Health) ‘Especially in rural areas, these mothers still have to work away from home and work in industrial zones. The ones who take care of the children are in fact their grandparents or other guardian.’ (local policymaker, Bắc Ninh) ‘In principle, the corporate workers and employees are entitled to six months of leave after birth. But the working class work as soon as they feel fine enough to go back.’ [local policymaker, Quảng Ngãi)
Vertical public administration and thickness of hierarchy	Role of government agency hierarchy and responsibility (Decree No. 100)	‘The Department of Maternal and Child Health and the Department of Legal Affairs - Ministry of Health were the two focal points to receive information about false advertising of dairy companies for punishment.’ (National Assembly)
	Confusion about the reporting process for violations of Decree No. 100 by frontline staff and lack of communication	‘Generally, there were some detections [violations], but what we [local organization] did was limited to sending them out. We had no idea how they were handled.’ (Women’s Union)
Networks/inter-organizational relationships	Importance of national-level implementing agencies visiting local sites to assess adherence to Decree No. 100, particularly for monitoring and enforcement efforts (e.g. in-person audits)	‘Inspectors of the Ministry of Health and the Ministry of Information and Communications also had to visit hospitals. I discovered that a physician wrote the name of the milk on a small piece of paper so that the children’s family could buy milk at the grocery store at a hospital gate. I immediately informed the Deputy Inspector General of the Ministry of Health, and at the same time informed the Director of the provincial Department of Health to warn physicians for their malpractice.’ (National Assembly)
	Strength of government relationships with Alive and Thrive and international organizations with expertise in maternal and child health	‘Since the WHO is a technical support organization, the cooperation with Alive and Thrive was very good given the Alive and Thrive project was specialized in feeding children. Alive and Thrive focuses very well on the feeding of children. The World Health Organization participated as a team together with UNICEF again to help the Ministry of Health in this area, so there was no problem.’ (World Health Organization) ‘When working with government partners, they supported us and there were also domestic champions at higher levels that supported us, supported the progress of the breastfeeding project, from national level to community level.’ (Alive & Thrive Vietnam)
	Limited role for external entities to contribute to policy implementation	‘Each project has its opening and ending time. We will have to step back anyway for the government to do it on their own. However, basically all ideas are required to be integrated into the existing system.’ (Save the Children Vietnam) ‘After the decree was issued, we should mention about the enforcement effort, including dissemination and monitoring. For example, monitoring newspapers to see if ads or articles are still illegal. We always kept an eye on enforcement and ensured the law was kept unchanged, and not attacked by any other forces.’ (Alive & Thrive Vietnam)
Implementing agency responses	Lack of funding for monitoring and enforcement of Decree No. 100	‘For the state agencies, instead of monitoring, the term “inspection and examination” is preferred in handling violations…we found this would be performed better if funds were available. If not, inspection and examination would be just half done.’ (Ministry of Health)
	Consideration of policy constraints on industry and private business	‘If we put too much pressure on businesses, they would withdraw. Now we encourage the development of private businesses. They have not started doing business but already have to take many duties.’ (MOLISA)
	Competing policies and programmatic priorities	‘We had to focus on other activities such as essential reproductive health care or new intervention models such as breast milk banks.’ (Alive & Thrive Vietnam) ‘The Institute for Legislative Studies had too many priorities, and the National Assembly also had more pressing issues.’ (Women’s Union)
	Limited knowledge and training among frontline workers and community members	‘People at the grassroots level, who would directly implement it, did not receive proper training.’ (Women’s Union) ‘A policy, no matter how promising it is, will be meaningless if not properly delivered at the local level. This is one lesson learned for all international organizations.’ (Ministry of Health)
	Costly and cumbersome reporting process for enforcement of Decree No. 100	‘Detectors were required to send photos of detection via express mail at their own expenses, while the subsequent claim process was time-consuming. The process required the refund to be paid to our account and the payment process needed to follow the norms set by the Ministry of Finance, which was painfully long. Women at the grassroots level needed to take pictures of violations and send them out. It was 2012 and smartphones were yet to be popular.’ (Women’s Union)
Attributes and responses of those affected by the policy	Limited reach of the extended maternity leave policy among marginalized populations (i.e. women who work in the informal sector and/or reside in rural, remote areas)	‘But beneficiaries are just civil servants and officials, and some employees. They also said that coverage was only moderate, not to all mothers. They asked us how many babies are born a year. I said that normally, for one year, there were about 1 million babies born, equivalent to 1 million mothers. Of these mothers, there were not many civil servants and contracted employees, but many rural workers.’ (MOLISA) ‘But the rural people do not have refrigerators, they go to the field to work in the sun, if they have milk, the milk does not preserve, how does one breastfeed? It is good if they put it in a cold bottle to breastfeed, but how can people know or have conditions to do so? So it is not easy for rural people to exclusively breastfeed.’ (local policymaker, Quảng Ngãi)
	Lack of nutrition education and misinformation about breastfeeding and breast milk substitutes	‘Young parents mainly take care of their children based on experience without thorough knowledge. Nutrition counseling is essential, especially to help parents realize the rich source of nutrition from home food, while they mostly pursue ready-made foods from foreign brands and rely too much on heavily advertised formula milk.’ (National Institute of Nutrition) ‘The misperception about formula milk has been around for a long time, one or two decades. The formula milk industry has dominated and changed the perception of people so returning to breast milk is very difficult.’ (Alive & Thrive Vietnam)
	Healthcare workers were receptive to enforcing Decree No. 100 in work settings	‘Medical workers better understood their role to avoid violating laws…when we introduced [the decree], which ruled that medical workers were not allowed to advertise dairy products or receive support from dairy companies, many did not know they were violating a term in law.’ (Save the Children)
External environment or policy context	Value of other policies that protect and promote the rights of women and children	‘Recently, I am pleased to say, in general, that regarding policies for women, Vietnam is one of the countries that strictly implement and sufficiently institutionalize international conventions, such as conventions on rights of women and children. There are international labor conventions, specific conventions for female workers, underage workers and policies for female workers. That policy has been embodied in laws, such as gender equality law, the labor law, social insurance law, health insurance policy and many other social assistance policies regulated in many official documents.’ (MOLISA)
	Influence of social context and norms that conflict with implementation of certain health policies	‘Protecting the rights of women and children works differently in each country. The first factor is culture and the other is the management ability of the country. For an example, asking children to buy cigarettes is prohibited in foreign countries, but in Vietnam, there are cases people doing it, although it is prohibited.’ (MOLISA)
	Influence of macroeconomic conditions that limit policy coverage	‘Because if we wanted to cover all [mothers], the number of people would be too high. I really wanted to do that, but because the national economic conditions were not strong enough, we did not have enough resources.’ (MOLISA)

Abbreviation: MOLISA = Ministry of Labor, Invalids, and Social Affairs.

a Study data mapped onto six of eight policy implementation determinants identified the integrated framework. We do not report on the Policy Formulation Process or Timing/Sequence due to lack of data on these topics. Framework source: Bullock *et al*. (2021).

### Determinant 1. Characteristics of the evidence-informed policy or practice

Policy precedence emerged as an important element of the policy context and blueprint to implement further restrictions on marketing of breast milk substitutes. Respondents spoke about the value of policy precedence (i.e. existing law) to support implementation of Decree No. 100. Some national-level respondents said having an existing policy (i.e. Decree 21) as the basis for Decree No. 100 facilitated their ability to rapidly implement the new policy because it was aligned with an existing policy context where processes and documentation already existed.

Challenges were noted about the compatibility of the extended maternity leave policy with the local context. Several respondents mentioned concerns with the policy’s compatibility with the reality and lived experiences of women who worked in informal labour markets, rural areas and/or worked far away from home. National- and local-level respondents also mentioned that the policy targeted mothers but did not impact guardians or caretakers (i.e. grandparents and child sitters) who primarily cared for infants and young children while parents worked.

### Determinant 2. Vertical public administration and thickness of hierarchy

Government entities identified as responsible for implementation of Decree No. 100 consisted of the Ministry of Health, namely, the Department of Maternal and Child Health, Department of Legal Affairs and Department of Inspections, in addition to the People’s Council. A key challenge was that frontline workers and community members involved in detecting and reporting violations were unsure of the reporting process, which may be related to a lack of communication in this hierarchy or standard operating procedure for reporting and response.

### Determinant 3. Networks/inter-organizational relationships

In terms of inter-governmental agency relationships and communication, respondents said it was important for national implementing agencies to visit local communities to monitor and enforce Decree No. 100. This allowed them to acquire local knowledge and communicate with other departments to strengthen implementation.

Most data for this determinant were related to relationships between government and non-government entities and international organizations. Specifically, the strength of these relationships and their coordination was said to facilitate policy revisions and implementation by offering evidence and coalescing partners around common policy goals. Respondents said it was challenging for non-governmental organizations to be involved in policy implementation because they did not have a clearly designated role beyond providing general support for implementation, primarily through monitoring and evaluation. Here, some respondents noted the importance of adequately equipping domestic agencies and authorities to take on policy implementation.

### Determinant 4. Implementing agency responses

An overarching challenge to implementation mentioned by several key informants was a lack of adequate funding for monitoring and enforcement activities. Other issues that constrained and impeded implementation were the potential influence on private businesses and competing policies and programmatic priorities. Respondents also said limited policy knowledge and training among frontline workers made it difficult to monitor and enforce Decree No. 100 in community settings, in addition to a reporting process that was cumbersome and costly for frontline workers and community members.

Policy precedence (described in Determinant 1) was noted as a facilitator by national-level respondents that helped implementing agencies effectively respond and carry out Decree No. 100 after its passage.

### Determinant 5. Attributes and responses of those affected by the policy

The extended maternity leave policy was said to have limited reach and impact among marginalized women who either worked informally or resided in rural or remote areas. At the local level, some respondents spoke about the lack of resources, like refrigeration, time or privacy, for working mothers to adequately pump and store breast milk during work hours. This was said to be a particular issue for women who worked in the fields in contrast to those employed in factories where these spaces were perceived to be more widely available.

Another challenge that undermined policy adherence and impact was the prevalence of myths and lack of education regarding exclusive breastfeeding and complementary feeding practices and nutrition for young children. A few actors mentioned the enduring impact of the breast milk substitute industry in propagating myths and misinformation about human milk and breast milk substitutes. Myths about formula milk held by a newborn’s grandparents or other people of influence (i.e. health workers and peers) were said to negatively impact a mother’s likelihood of initiating or continuing to breastfeed.

A key facilitator to the implementation of Decree No. 100 consisted of healthcare personnel who were said to be receptive to reducing the promotion of breast milk substitutes in healthcare settings.

### Determinant 6. External environment or policy context

In terms of the broader policy environment, respondents named several country policies that protected and promoted the rights of women and children, providing an important basis for adoption and implementation of the new policies. Existing policies and regulations were said to protect female workers in the labour market (particularly expectant mothers), promote children’s health and well-being, and provide social assistance. According to the respondents, international labour conventions for female workers and underage workers were embodied in Vietnam’s gender equality law, labour code, social insurance law and health insurance policy. Policies to protect children were further subcategorized into policies related to infants (<12 months), young children (<36 months) and school-aged children.

An implementation challenge in the external environment was the conflict between existing socio-cultural norms/practices and policies as well as the influence of macroeconomic conditions that limited the coverage scope of the extended maternity leave policy.

## Discussion

Our qualitative study investigated the implementation of further restrictions on marketing of breast milk substitutes (Decree No. 100) and extended maternity leave policy in the Labour Code in Vietnam—identifying opportunities to improve implementation of these policies. Key informants spoke highly of these two policies and said they functioned synergistically to promote an enabling environment for women to initiate and continue breastfeeding, thus advancing the health of women and young children in the country.

Our results on the importance of policy precedence speak to the value of understanding a policy’s established historical and legal context in addition to the external environment and policy context (i.e. political and social climate) identified in the integrated framework (Bullock *et al.*, 2021). Policy precedence emerged as an important determinant and model for Decree No. 100 that facilitated implementation. While policy precedence is influenced by other determinants, including the external environment or policy context, it is not the same because Decree No. 21 falls within the same maternal and child health policy subsystem as Decree No. 100. This construct merits further study in the policy sciences and public policy frameworks since prior research examining state policy adoption in the USA found that policy precedence was an advocacy strategy used by a coalition (Payán *et al.*, 2017), indicating its significance across policymaking processes.

While policy adoption has garnered more attention in recent years, this study seeks to advance scholarship on maternal and child health policy actors, processes and contexts related to ‘implementation’. Policy implementation actors identified in this study are similar to those mentioned in existing literature that report UNICEF and formula companies are at the centre of the international actor network, and government institutions (e.g. National Institute of Nutrition) are central to the domestic policy actor network in Vietnam (Harris *et al.*, 2017). Our findings elaborate on the role of these policy actors and the constraints they encounter during early implementation. Our study also highlights the importance of specifically delineating and reviewing the role of different actors throughout the policymaking process. For example, while external entities and partners like Alive and Thrive can play an important role in policy advocacy (Harris *et al.*, 2017), the extent of their involvement may be limited during implementation since national and local government institutions are often exclusively tasked with policy enforcement and monitoring activities. Additional research is needed to investigate power dynamics and communication between implementation actors—including their social networks (Rasheed *et al.*, 2017) and the influence of their political and social contexts (Erasmus *et al.*, 2014).

This article calls attention to the critical role of frontline workers in bureaucracies and local implementing agencies who serve as the street-level bureaucrats, defined as those in the public sector tasked with dispensing benefits or allocating sanctions/enforcement duties (Lipsky, 2010). While these actors are key to understanding how health policy is being implemented in LMICs, they are often overlooked in policy research or are hidden from the public (Kamuzora and Gilson, 2007). The results shed light on problems with detection and reporting of violations of Decree No. 100 in Vietnam, possibly due to limited training for frontline workers and community members, a lack of communication between implementing agencies and actors, and a challenging local reporting process. The receptiveness of health personnel to reduce promotion of breast milk substitutes in healthcare settings is promising and highlights an opportunity for further investment and collaboration with these willing supporters to enhance implementation. Recommendations for improvement include simplifying the reporting and response process for violations of Decree No. 100 among frontline workers and community members in non-healthcare settings and enhancing local-level knowledge and mechanisms for monitoring and enforcement of violations for both policies. Examining the behaviour (individual and collective), resources available and conditions of street-level bureaucrats tasked with enforcement of maternal and child health policy is a valuable avenue of inquiry to advance the science of policy implementation.

Findings on the determinants of policy implementation can be used to inform other strategies to fully implement policies to support maternal and child health in LMICs. Data suggest a lack of compatibility between the extended maternity leave policy and local context for women in the country as well as the limited reach among marginalized women in rural areas or those informally employed. Like Vietnam, working women employed in informal labour markets elsewhere may not be covered by maternity leave protections (Chai *et al.*, 2018; Rasheed *et al.*, 2017). If extending maternity leave protections is not politically or economically feasible, alternatives can include providing maternity leave cash transfers (Siregar *et al.*, 2021). A remaining area for research is to measure potential discrimination and stigma from employers against reproductive-aged or pregnant women (as well as mothers with young children) in response to maternity protections.

Lack of funding was a primary barrier to general policy implementation, which raises concern about the sustainability and long-term impact of external donor funds on domestic agency function and expectations. Prior studies report key challenges to IYCF policy implementation include lack of resources (funding and personnel), lack of funding plans and lack of enforcement capacity, contributing to weak policy implementation and effectiveness (Harris *et al.*, 2017; Rasheed *et al.*, 2017). It is possible that the loss of technical support and general finances to sustain Alive and Thrive Initiative activities (Ouedraogo *et al.*, 2021; Sanghvi *et al.*, 2016) may have impacted implementation since several of these agencies are the same as those who helped to deploy the Alive and Thrive Initiative. Having sufficient monitoring and evaluation infrastructure and training is also necessary to strengthen the IYCF policy context (Avula *et al.*, 2017; Baker *et al.*, 2013; Jean *et al.*, 2013; Sanghvi *et al.*, 2016; 2013).

Supportive practices and policies can boost policy implementation. Increased awareness and utilization of innovative interventions to facilitate breastfeeding—like the first human milk bank established in Vietnam in 2017 (Tran *et al.*, 2021) and Decree No. 145/2020/ND-CP, which has a labour stipulation that employers with at least 1000 female employees must have a room for breast milk collection and storage (effective 1 February 2021) (Truong, 2021)—are complementary efforts for a supportive policy environment. Breastfeeding counselling (Keats *et al.*, 2021; Rollins *et al.*, 2016; Tuan *et al.*, 2014), educational IYCF videos (Schneider *et al.*, 2021) and mass media campaigns (Nguyen *et al.*, 2017) in areas with higher rates of violations can also help dispel myths and improve local breastfeeding rates. Having a robust monitoring, evaluation and reporting system for violations would be fundamental to inform decisions about where to target programmes and resources. Community-support IYCF groups are a potentially feasible and effective intervention to reach women in remote and rural areas (Nguyen *et al.*, 2021).

Important avenues of inquiry for future investigation include maternal and child health policy surveillance research to obtain a comprehensive sense of the policy environment and to support legal epidemiology research on the topic. In reality, multiple policies are often simultaneously in effect and at different stages of the policy cycle. A noted limitation of policy effectiveness research is that it may not account for the implementation of other policies or interventions (Chai *et al.*, 2018). Researchers and funders should also invest in cross-country comparisons of policy implementation using the integrated theoretical framework using mixed methods and in-depth qualitative approaches. While cross-country comparisons are time- and resource-intensive (Walt *et al.*, 2008), it would be a worthwhile next step in addition to policy surveillance methods to advance the emerging field of policy implementation in LMICs.

### Strengths and limitations

This study has several noteworthy strengths. This is among the first studies to leverage the integrated framework of the policy implementation process, which is a step towards addressing the dearth of theory used in policy implementation research (Erasmus *et al.*, 2014) and policy analysis research (Walt *et al.*, 2008). A second strength is the use of qualitative data to elaborate on implementation processes (thus providing rich local context and experiences from various perspectives), which can be difficult to examine due to social, political, bureaucratic, language and cultural differences. Collecting data in 2017 allowed for a sufficient lag time for implementation after policy adoption without being excessively long after adoption to avoid recall bias or the influence of other maternal and child health policy debates (Walt *et al.*, 2008). Our inclusion of two policies is another strength as it encourages other researchers to look beyond a singular policy or law when examining implementation or impact.

A study limitation is the potential selection bias due to the exclusion of other policy stakeholders (e.g. breast milk substitute industry, employers and new mothers) who may have been involved in the policymaking process or impacted by the implementation of these policies. The involvement of the private sector, particularly of for-profit organizations, and the impact of public–private partnerships in health policy should be examined in future work (Walt *et al.*, 2008). Another limitation is that policy implementation was not the exclusive focus of the interview tool, and we were not able to conduct in-depth interviews about implementation due to resource limitations. We did not specifically ask respondents about the policy formulation process or timing/sequence, which is potentially why the data did not map onto these two determinants from the integrated framework.

## Conclusion

This study provides valuable insights into the determinants of implementing an extended maternity leave policy in the Labour Code and further restrictions on marketing of breast milk substitutes (Decree No. 100) in Vietnam, revealing the importance of local street-level bureaucrats tasked with enforcement and social context and norms around breastfeeding. Recommendations include strengthening the policy environment by offering protections to women in the informal labour sector in addition to supportive robust practices (i.e. breastfeeding counselling). Our results also back the following recommendations from a report on the Code to allocate sufficient budgets and human resources for full implementation, establish robust and sustainable monitoring and enforcement mechanisms of these policies, and educate healthcare workers regarding their responsibilities and role in the implementation and promotion of breastfeeding (World Health Organization, 2020). Policy implementation research is a promising avenue to identify and tailor strategies and recommendations to enhance the implementation of policies to support optimal IYCF practices.

## Supplementary Material

czab116_Supp

## Data Availability

The data underlying this article cannot be shared publicly since this request was not included in the informed consent process and to protect the privacy of respondents. Additional interview excerpts will be shared upon reasonable request to the corresponding author.
