# Supplementary material for: Implementation of two policies to extend maternity leave and further restrict marketing of breast milk substitutes in Vietnam: a qualitative study
Source: Health Policy Plan. 2021 Sep 18;37(4):472–82. doi: 10.1093/heapol/czab116 (PMC9006067; doi:10.1093/heapol/czab116)
Supplement: czab116_Supp [file czab116_supp.zip › Supp data. interview guides.docx]

**Guide for Semi-Structured Key Informant Interviews**

**Viet Nam, national level**

***INSTRUCTIONS TO INTERVIEWERS***

*Important information about using this interview guide:*

1. This guide should be used with all national-level partners (i.e. government, funders, implementing partners at the central level).
2. ALL questions contained in this guide must be asked during the interview EXCEPT where stated otherwise.
3. Read this script before beginning the interview. Check the appropriate boxes below before proceeding.

*Thank you for your willingness to participate in an interview about infant and young child feeding (IYCF) programs in Viet Nam, including about the Alive & Thrive (A&T) initiative. These questions are being asked as part of a learning exercise about A&T implementation and sustainability. There are no right or wrong answers to these questions. Your name will not be linked to any information that you individually provide. This is not a test of your knowledge, if you do not know an answer to a specific question that is okay. You can skip any questions you want, and you can end our conversation at any time. I will take notes, and if it’s okay with you, I would like to audio record, so I can focus more on our discussion and fill in my notes later. If you don’t want me to record or want to stop the recording at any time, that is completely okay, just let me know. Do you have any questions before we get started?*

Do I have your permission to begin this interview? O Yes O No (THANK RESPONDENT & STOP HERE)

Do I have your permission to audio record? O Yes O No (TURN OFF AUDIO RECORDER)

*Interview with (check one):*

O A&T global leadership O USG representative in-country (i.e. CDC or USAID)

O A&T implementing partner (specify) ______________________________

O Central-level government representative (specify) ___________________________

O Other funding agency (specify) ________________________

O Other (specify) _____________________________

**Introduction**

1. What would you consider to have been the most important successes of A&T, and why? [Probe for concrete examples of positive elements.]
2. How, if at all, have IYCF activities changed since the A&T program ended in 2014? [Probe for details about reach/scope, quality, impacts. Probe for any new IYCF policies that were implemented since A&T ended.]
3. What, if any, plan was in place to continue IYCF activities after the A&T program ended? How was the plan implemented in reality after the project conclusion?

**Implementation**

1. What would you consider to be the three most difficult challenges that you encountered during A&T implementation and why? How did these affect program implementation? [Probe for concrete examples of barriers and challenges faced to implementation.]
2. What would you consider to be the three main factors that have facilitated A&T implementation for you? [Probe for concrete examples of people, systems, environments, etc. that facilitated A&T implementation.]

**Other programs**

1. To your knowledge, have other programs adopted A&T's IYCF tools and specific activities? [probe for specific examples]
2. To your knowledge, how have other programs influenced A&T tools and activities?
3. Are there collaborative initiatives that A&T worked with other programs to promote and are these activities continuing?

**Leadership**

1. What are your overall impressions about leadership of IYCF activities in Viet Nam? [Probe for specific leadership challenges, if any.]
2. Can you tell me more about who was engaged in the scaling up the A&T supported program after the project conclusion? (Funding agency, implementing agency)

**Funding**

1. Please describe the main challenges (if any) faced in continuing to fund IYCF activities in Viet Nam. [Probe: how sustainable is the funding for IYCF activities?]

**Monitoring**

1. How well is the monitoring system working/functioning? [Probe: What is working well and why; what is not working well and why? Probe about monitoring for quality, scope, fidelity, impact.]

**Best practices & moving forward**

1. A&T is now being implemented in additional countries. Based on your overall experiences with A&T, what advice would you have for new partners or countries joining A&T in this new phase? [Probe: What are best practices that should be supported? What aspects do you feel did not work well & should be changed as the program moves into these new countries?] What are influencing factors to take the A&T supported IYCF to a sustainable application for a country?
2. What else do we need to know about A&T program implementation and sustainability that has not been covered in this interview?

**Guide for Semi-Structured Key Informant Interviews**

**Viet Nam, sub-national level**

***INSTRUCTIONS TO INTERVIEWERS***

*Important information about using this interview guide:*

1. This guide should be used with all sub-national-level partners (i.e. district government, implementing partners at the local level).
2. ALL questions contained in this guide must be asked during the interview EXCEPT where stated otherwise.
3. Read this script before beginning the interview. Check the appropriate boxes below before proceeding.

*Thank you for your willingness to participate in an interview about infant and young child feeding (IYCF) programs in Viet Nam, including about the Alive & Thrive (A&T) initiative. These questions are being asked as part of a learning exercise about A&T implementation and sustainability. There are no right or wrong answers to these questions. Your name will not be linked to any information that you individually provide. This is not a test of your knowledge, if you do not know an answer to a specific question that is okay. You can skip any questions you want, and you can end our conversation at any time. I will take notes, and if it’s okay with you, I would like to audio record, so I can focus more on our discussion and fill in my notes later. If you don’t want me to record or want to stop the recording at any time, that is completely okay, just let me know. Do you have any questions before we get started?*

Do I have your permission to begin this interview? O Yes O No (THANK RESPONDENT & STOP HERE)

Do I have your permission to audio record? O Yes O No (TURN OFF AUDIO RECORDER)

*Interview with (check one):*

O A&T implementing partner (specify) ______________________________

O Local-level government representative (specify) ___________________________

O Other funding agency (specify) ________________________

O Other (specify) _____________________________

**Introduction**

1. How, if at all, have IYCF activities in your region changed since the A&T supported program ended in 2014? [Probe for details about reach/scope, quality, impacts.]
2. What, if any, plan was in place to continue IYCF activities after the A&T supported program ended?

What do you think about the quality of MTBT franchise at provincial level, in comparison to those at district and commune level? What are the key influencing factors to the good quality of performance?

**Implementation**

1. What would you consider to be the three most difficult challenges that you encountered during A&T implementation and why? How did these affect program implementation? [Probe for concrete examples of barriers and challenges faced to implementation.]
2. What would you consider to be the three main factors that facilitated A&T implementation for you? [Probe for concrete examples of people, systems, environments, etc. that facilitated implementation.]

**Adaptation**

1. How, if at all, have the MTBT franchises changed since the A&T program ended in 2014? Why were these changes made? [Probe: How, if at all, are the franchises combined with other health programs or interventions?]

**Leadership and partnership**

1. How, if at all, did Alive and Thrive supported programming leadership change after the conclusion of the project? Why were these changes made?

**Funding**

# After the project conclusion, how did the provincial agencies provide funding to the A&T supported activities?

**Monitoring**

1. How do you know if IYCF activities are being implemented as planned? How do you know if they are having the intended impact?
2. How well is the monitoring system working/functioning? [Probe: What is working well and why; what is not working well and why? Probe about monitoring for quality, scope, fidelity, impact.]

**Best practices & moving forward**

1. A&T is now being implemented in other province in Vietnam. Based on your overall experiences with A&T supported program, what are the top three lessons that you would share as advice for new partners or countries joining A&T in this new phase? [Probe: What are best practices that should be supported? What aspects do you feel did not work well & should be changed as the program moves into these new provinces?]
2. What else do we need to know about A&T program implementation and sustainability that has not been covered in this interview?
